# Changes in unhealthy food consumption among vulnerable elementary-aged children in the United States during the COVID-19 pandemic: a serial cross-sectional analysis

**DOI:** 10.3389/fnut.2024.1468767

**Published:** 2024-09-25

**Authors:** Nivedhitha Parthasarathy, Nalini Ranjit, Ru-Jye Chuang, Christine Markham, Mike Pomeroy, Jacqueline Noyola, Deepali K. Ernest, Shreela V. Sharma

**Affiliations:** ^1^Department of Health Promotion and Behavioral Sciences, University of Texas Health Science Center at Houston, School of Public Health, Houston, TX, United States; ^2^Michael and Susan Dell Center for Healthy Living, Department of Health Promotion and Behavioral Sciences, University of Texas Health Science Center at Houston, School of Public Health in Austin, Austin, TX, United States; ^3^Center for Health Equity, Department of Epidemiology, University of Texas Health Science Center at Houston, School of Public Health, Houston, TX, United States; ^4^Brighter Bites, Houston, TX, United States

**Keywords:** diet, unhealthy food, children, COVID-19 pandemic, public health nutrition

## Abstract

**Objective:**

Several studies suggest that during the early pandemic, amidst socioeconomic instability, children from underserved families were more likely to resort to consuming cheaper, lower-quality foods with longer shelf lives. This study investigated the change in unhealthy food consumption across different phases (pre, early, mid) of the COVID-19 pandemic, and whether the strength of association between unhealthy food consumption and household socioeconomic disadvantage (HSED) varied across phases of the pandemic.

**Methods:**

This study utilized serial cross-sectional data collected from low-income families enrolled in a school-based food co-op, Brighter Bites. Secondary data analysis included 5,384 surveys from families who had complete data: 3422 pre-pandemic, 944 from the early pandemic, and 1,018 mid-pandemic. Outcome measures included sugary food intake and convenience/fast food intake, each of which was operationalized as a scale using pre-validated items from the surveys. HSED was operationalized as a composite of parent employment, parent education, food insecurity, and participation in government programs; responses were categorized into low, medium, and high HSED groups for analysis. We examined the interaction between HSED and time period to explore how HSED and its association with dietary measures changed over the course of the pandemic.

**Results:**

A significant linear negative trend, i.e., a decrease in consumption from pre-to-mid-pandemic period was seen in sugary food intake (*p* < 0.001), but not in convenience food intake. In data pooled across time-periods, both sugary food and convenience/fast food consumption were inversely associated with HSED, and low unhealthy food consumption was observed among high-disadvantage groups. No significant interaction between time period and HSED was observed on either scale. However, the post-regression estimates from the adjusted interaction model showed a significant association between convenience/fast food consumption and HSED in pre- and mid-pandemic periods.

**Conclusion:**

The study highlights the nuances of socioeconomic dynamics on the diet behaviors of children from low-income families during a natural disaster.

## Introduction

Unhealthy food consumption contributes to a plethora of chronic diseases in the United States (U.S.) (1, 2); nearly half the elementary-aged children (6–11 years) consume inferior quality diets and excess junk food compared to their peers in other age groups (3–5). During the early phase of the COVID-19 pandemic, social measures like lockdowns/school closures implemented to curb transmission of the SARS-COV2 virus, worsened diet quality among families with children (6, 7). Longitudinal studies conducted on diverse U.S. populations reported the highest increase in eating disorders and BMI in this age group (8). As the elementary school age is considered a critical period in the development of chronic diseases that track into adulthood, it is vital to identify any detrimental diet changes that may have occurred during the pandemic, among this population (9).

Socioeconomic status (SES) has emerged as a crucial determinant of diet and nutrition quality, and their downstream health consequences; as families with lower SES often face barriers such as limited financial resources, reduced access to healthy foods, and greater reliance on cheaper, calorie-dense options, which can lead to poorer diet quality and subsequent health issues (10). Particularly during times of crises, families experiencing poverty are more vulnerable to macroeconomic fluctuations (11, 12). Along with the ongoing challenges that low-income families face, the added burden of food insecurity further exacerbates their situation, creating a cumulative socioeconomic disadvantage (13). During the pandemic, millions of Americans experienced pandemic-related job losses and financial setbacks, resulting in a three-fold increase in the prevalence of food insecurity in the U.S. (14). Low-income families with children, especially those belonging to racial minority groups, were disproportionately affected by interruptions in food supply chain, government assistance programs, and nutritious school meals. Overburdened with financial insecurity and loss of access to reliable food sources, these families were forced to choose affordability over quality of food (15–19). Studies conducted during the early pandemic reported an increase in the purchase and consumption of sugary drinks, chips, ready-to-eat meals, and a decrease in fresh produce, among children belonging to such vulnerable families (19, 20). However, a majority of these studies did not include objective control data from pre-pandemic time for comparison. Furthermore, as schools reopened for hybrid learning in 2021, USDA introduced new waivers in school meal programs, such as meal-time and meal pattern flexibility to ensure continued access to school meals (21–23). There is little information on how elementary-aged children’s diet changed with the re-opening of schools and economic instability experienced in the U.S. at that time, with objective comparative data (24, 25). It is of particular interest to understand how children’s dietary behaviors were impacted by rapidly evolving government mandates and employment situations, especially since this knowledge provides critical insights into the immediate health impacts of the pandemic and serves as a foundation for evidence-based disaster preparedness strategies.

Therefore, this study aims to investigate changes in unhealthy food consumption across different phases (pre, early, mid) of the COVID-19 pandemic. The second objective is to examine the strength of associations between unhealthy food consumption and household socioeconomic disadvantage, and determine whether the magnitude of these associations varied across phases of the pandemic.

## Materials and methods

### Research design and study participants

This study utilized serial survey-based cross-sectional data collected between 2018 and 2022 from Brighter Bites, a theory-grounded school-based food co-op health intervention serving low-income families across the U.S. (26). Brighter Bites is a 501c3 non-profit organization that implements school-based health promotion programs in Texas (Houston, Austin, Dallas, San Antonio); Florida (Southwest Florida); New York (New York City); Washington, D.C.; California (Salinas, Los Angeles, Bakersfield); and Arizona (Phoenix). The organization provides free fresh produce and implementation support of nutrition education at schools that predominately serve children from low-income families for 3 years in a row, to mitigate food insecurity and improve dietary habits (16, 27–29). Each school year, at the beginning of the fall semester, before the Brighter Bites program began, informed consent was obtained, and electronic surveys were sent to parents to obtain data. Survey completion and participation in the study was voluntary, and all participants provided informed consent. The University of Texas Health Science Center at Houston (UTHealth Houston) is the evaluation partner for Brighter Bites. As a part of a data sharing agreement, deidentified data were shared with UTHealth for analysis. The UTHealth Committee for Protection of Human Subjects approved the parent study (HSC-SPH-15-0752).

The current analysis is based on four of these annual baseline parent surveys (2018–2019, 2019–2020, 2020–2021, 2021–2022) from seven areas (Houston, Dallas, Austin, Southwest Florida, Washington, D.C., Salinas, and Bakersfield). The response rate for each year is as follows: 2018–28.1%, 2019–27.9%, 2020–33%, 2021–16.8%. Parent and child data from 5,384 surveys (self-reported data from the parents); 3,422 during the pre-pandemic period (fall 2018, 2019), 944 from the early pandemic period (fall 2020), and 1,018 from the mid-pandemic period (fall 2021) were included in the analysis.

### Measures

#### Children’s unhealthy food consumption

Nine pre-validated items (30) were identified from the parent survey that captured the frequency of consumption of the following unhealthy items in the week prior to survey completion – sugar-sweetened beverages (SSB) (3 items), sugary cereals, frozen desserts, fried potatoes, chips, heat-and-serve meals, and meals from the restaurant. The responses for all the items were captured on a Likert scale (never in a week – every day in a week). For this study, dietary items were broadly classified into two main categories based on the Family Life, Activity, Sun, Health, and Eating (FLASHE) study data guide (30). (1) Sugary foods including Sugar-Sweetened Beverages (SSB), sugary cereals, and frozen desserts, and (2) Convenience/Fast-food including fried potatoes, chips, heat-and-serve meals, and meals from restaurants. These categories represented the two most consumed unhealthy food groups during the pandemic period (31–34). The possible score for sugary foods ranges between 0 to 20, and the possible score for the convenience/fast-food category ranges between 0 and 16. In both cases, higher scores represented a higher frequency in the consumption of that food group.

#### Assessment of household socioeconomic disadvantage

Socioeconomic disadvantage is a multidimensional construct that cannot be captured by single item scales (10). Especially in low income populations, where income sources are irregular and not reliably reported, alternative measures need to be utilized to capture variation of socioeconomic status. We utilized a composite household socioeconomic disadvantage measure from literature, which encompassed indicators both stable and those that were relatively exacerbated by the pandemic (35). Based on literature (10, 35), we utilized four variables: parent employment, parent education, food insecurity, and participation in government assistance programs to create the composite measure – household socioeconomic disadvantage.

In our dataset (pooled over time period), when analyzed independently, we noticed that almost all components of HSED were significantly associated with both sugary food (*p* < 0.05) and convenience/fast food (p < 0.05), except for food insecurity with convenience/fast food scale (*p* = 0.643); the strongest association was observed for food insecurity with sugary food scale (Coeff: 0.22; *p* = 0.004), and for parent education with convenience/fast food scale (Coeff: 0.36; *p* = <0.001). Although food insecurity was not a significant predictor, we included it in our composite HSED measure due to its empirical association with diet behavior, and its relevance to the COVID-19 pandemic.

Information regarding each component of household socio-economic status was captured using appropriate single-item scales (10, 36). Information regarding parent education was categorized into 0: never – attended middle school; 1: attended/graduate high school; and 2: attended/graduated college; employment status of the parent was categorized as 0: involuntarily unemployed; 2: voluntarily unemployed; and 2: employed. Food insecurity was measured using the Hunger Vital Sign questionnaires, and individuals who selected “sometimes true” or “often true” for either question were classified as food insecure (37). Participation in any government assistance programs (WIC, SNAP, Double Dollars, Medicaid, Medicare, CHIP, free/reduced meals at school) were categorized as not participating or participating. Using the above information, we created a composite measure of household socio-economic disadvantage, guided by similar work in prior literature (10, 35). The summative composite score ranged from 0 to 8 representing decreasing order of household socioeconomic disadvantage (HSED). The scores were grouped into high (score 0–2), medium (score 3–5), and low (score 6–8) disadvantage groups for analysis. Additionally, each HSED component was examined independently in relation to the outcomes, to provide insight into their role as individual predictors of unhealthy food consumption.

#### Effect modifier

We examined whether the phase of the pandemic modified the relationship between socioeconomic disadvantage and dietary measures. Three phases were identified: pre-pandemic (fall 2018 to fall 2019), early pandemic (fall 2020), and mid pandemic (fall 2021). This categorization of corresponded to macro-socioeconomic changes that occurred in the U.S. during this time. Macroeconomic changes in the early pandemic phase (March 2020 – March 2021) included increases in unemployment, the introduction of government fiscal response (Families First Act; CARES act; SNAP and WIC waivers) while macrosocial changes include school closures, and halting of school lunch programs. The macroeconomic environment of the mid pandemic phase (March 2021 – May 2022) was characterized by high inflation, decline in unemployment, extension of benefits, and macrosocial changes such as school reopening, adapted school nutrition programs (e.g., waivers in NSLP) (21, 23–25, 38).

#### Covariates

Data on child’s grade (K-5), gender (male, female/), race (Black/African American, Hispanic/Latino/Mexican American, White/Caucasian, Asian, Native Hawaiian/Pacific Islander, American Indian/Alaskan Native, Other), number of children at home, and number of people at home were collected in the surveys. Beneficial food consumed in the week before the survey was captured in the following items – 100% fruit juice, water, fresh fruits, vegetables, and other non-fried vegetables. These variables were combined to form a beneficial food scale (30).

## Statistical analysis

Schools that were only in the first year of the Brighter Bites program, (i.e., those who had not received any component of the intervention at the time of the baseline survey), were included in the analysis. Means and standard deviations were reported for continuous measures while proportions were reported for. Categorical variables. The changes in unhealthy food consumption over time (time period 0 = pre-pandemic; 1 = early pandemic; 2 = mid pandemic) were reported using p for linear trend. Mixed effects regression models were built using pooled data from 2018 to 2021 and were used to examine the strength of the association of unhealthy food consumption with household socioeconomic disadvantage. Covariates were selected for analysis using Wald tests. Univariable analysis was conducted to determine the unadjusted associations between the outcome and each covariate. Next, based on statistically significant bivariate associations with the outcome, and modification of regression coefficients by 10%, we identified that child’s race and beneficial food scale served as potential confounders and adjusted for them in our analysis. We also ran initial models including location fixed effects, but none of these effects was significant, hence they were discarded. The overall fit of the models was determined using likelihood ratio tests. Additional tests for multicollinearity included checking the variance inflation factor (VIF). No multicollinearity was detected.

To assess changes in this association over time, an interaction term between household socioeconomic disadvantage and time period was added to the model. Post-regression estimates including predicted means and pairwise comparison of effects were examined to quantify the independent and joint effects of household socioeconomic disadvantage and time period. Mixed models accounted for school-level clustering. Statistical significance was set at *p* < 0.05. All analyses were performed using STATA Version 17.0 (College Station, TX: Stata Press. StataCorp. 2019).

## Results

Overall, 5,384 participants were included in the final analysis; this includes complete data from 3,422 surveys during the pre-pandemic period (fall 2018, 2019), 944 surveys from the early pandemic period (fall 2020), and 1,018 surveys from the mid-pandemic period (fall 2021). The sociodemographic data of the participants included in the analysis are presented in Table 1. Mothers (92.4%) completed the majority of the surveys. In all three time periods, a majority of the children studied in grades 3–5 (pre: 39.7%; early: 47.9%; mid: 45.4%), and belonged to Mexican American/Hispanic/Latino race/ethnicity (pre: 83.7%; early: 82.4%; mid: 81.5%). According to parent demographic data, a majority of the parents either attended or completed high school (pre: 53.5%; early: 57.9%; mid: 52.1%), and were voluntarily unemployed (pre: 50%; early: 48.2%; mid: 55.6%). More than three-quarters of the parents reported participating in government assistance programs (pre: 87.9%; early: 93.6%; mid: 92.3%), and approximately quarter of them reported food insecurity (pre: 26.7%; early: 15.3%; mid: 25.4%). A majority of the participants were at greatest disadvantage; more than one-third belonged to high socioeconomic disadvantage groups during all the time periods (pre: 38.6%; early: 42.6%; mid: 44.1%).

**Table 1:** Sociodemographic summary of the families included in the analysis (Brighter Bites pre-fall parent surveys 2018–2022).

Measure		Time period			
		Pre-pandemic	Early pandemic	Mid-pandemic	*p*-value
Child grade *N* (%)	Kindergarten	745 (21.8%)	178 (18.9%)	158 (15.5%)	**<0.001** ^1^
	Grades 1–2	1,319 (38.5%)	314 (33.3%)	398 (39.1%)	
	Grades 3–5	1,358 (39.7%)	452 (47.9%)	462 (45.4%)	
Child gender *N* (%)	Male	1,663 (49.5%)	475 (50.7%)	483 (48.1%)	0.57^2^
	Female	1,695 (50.5%)	462 (49.3%)	522 (51.9%)	
Child race *N* (%)	White/Caucasian	170 (5.1%)	16 (2%)	63 (8%)	0.58^1^
	Mexican-American/Hispanic/Latino	2,767 (83.7%)	666 (81.4%)	647 (82.5%)	
	Black/African-American	256 (7.7%)	85 (10.4%)	47 (6%)	
	Other	113 (3.4%)	51 (6.2%)	27 (3.4%)	
Number of children at home Mean (SD)		2.6 (1.2)	2.7 (1.2)	2.5 (1.1)	0.03^3^
Number of adults at home Mean (SD)		1.5 (1.1)	2.5 (1.2)	2.5 (1.1)	0.35^3^
Individual who answered the survey *N* (%)	Mother	3,163 (93.1%)	873 (92.6%)	939 (92.4%)	0.45^2^
	Other	236 (6.9%)	70 (7.4%)	77 (7.6%)	
Parent employment status *N* (%)	Involuntarily unemployed	258 (8.2%)	141 (17.8%)	71 (9.3%)	**<0.001** ^1^
	Voluntarily unemployed	1,578 (50%)	382 (48.2%)	423 (55.6%)	
	Employed	1,317 (41.8%)	269 (34%)	267 (35.1%)	
Parent education status *N* (%)	Never attended school – 8th grade	530 (16.7%)	140 (17.7%)	133 (17.6%)	0.18^1^
	9th grade – GED	1,699 (53.5%)	457 (57.9%)	395 (52.1%)	
	Attended/Graduated college	949 (29.9%)	192 (24.3%)	230 (30.3%)	
Participation in government assistance programs (Yes) *N* (%)		2,964 (87.9%)	822 (93.6%)	812 (92.3%)	**<0.001** ^2^
Food insecurity (Yes) *N* (%)		845 (26.7%)	127 (15.3%)	210 (25.4%)	**0.001** ^2^
Household socioeconomic disadvantage^a^	Low	1,211 (35.6%)	254 (28.9%)	244 (27.6%)	**<0.001** ^1^
	Medium	877 (25.8%)	251 (28.5%)	251 (28.4%)	
	High	1,312 (38.6%)	375 (42.6%)	390 (44.1%)	

Bold indicates statistical significance; ^1^Jonckheere-Terpstra Test for trend; ^2^Cochran-Armitage Trend Test; ^3^Linear Trend Test; a Composite measure that include parent employment, education, food insecurity, and participation in government assistance programs.

### Changes in unhealthy food consumption over the course of the pandemic

A significant negative trend was observed in the sugary foods scale, indicating a decrease in the consumption of sugary food from the pre- to mid-pandemic period (*p* < 0.001). For the convenience/fast food scale, we noticed a slight increase in consumption from the pre- to early pandemic, followed by a decrease to the mid-pandemic. No significant linear trend was observed (*p* = 0.57; Table 2).

**Table 2:** Serial cross-sectional changes in child unhealthy food consumption through the course of the pandemic (Brighter Bites 2018–2022).

Measure	Time period			*p*-value^4^
	Pre pandemic (2018, 2019) Mean (SD)	Early pandemic (2020) Mean (SD)	Mid pandemic (2021) Mean (SD)	
Sugary foods^1^	5.3 (2.55)	5.06 (2.69)	4.87 (2.51)	**<0.001**
Sweetened fruit drinks and teas^3^	1.35 (0.93)	1.34 (0.94)	1.29 (0.89)	
Soda^3^	0.86 (0.7)	0.76 (0.7)	0.79 (0.67)	
Sports drinks^3^	0.78 (0.82)	0.66 (0.79)	0.64 (0.76)	
Sugary cereals^3^	1.30 (0.95)	1.28 (0.95)	1.16 (0.88)	
Frozen desserts^3^	1.08 (0.75)	1.13 (0.75)	1.10 (0.77)	
Convenience/fast foods^2^	4.03 (1.99)	4.07 (2.14)	3.97 (1.96)	0.57
Fried potatoes^3^	1.10 (0.66)	1.13 (0.67)	1.1 (0.61)	
Potato chips^3^	1.26 (0.76)	1.22 (0.76)	1.27 (0.79)	
Heat and serve food^3^	0.69 (0.86)	0.86 (0.80)	0.69 (0.83)	
Food from restaurant^3^	1.02 (0.60)	0.93 (0.61)	0.98 (0.58)	

Significance at *p* < 0.05; Bold indicates statistical significance; ^1^Score range for sugary foods scale = 0–20; ^2^Score range for convenience/fast food scale = 0–16; ^3^Score range for all component variables = 0–5 (never, 1–2 times, 3–4 times, 5–6 times, and every day); ^4^Linear test for trend.

Mixed effect models were used to estimate the magnitude of change in sugary foods and convenience/fast foods scale during the pandemic as compared to pre-pandemic period. As compared to the pre-pandemic period, sugary food consumption decreased in the early pandemic period (*β* = −0.29; 95% CI: −0.56, −0.02; *p* = 0.032), and in the mid-pandemic period (*β* = −0.51; 95% CI: −0.76, −0.25; *p* = <0.001), after adjusting for confounders. Convenience/fast food consumption showed only slight and non-significant decreases in the early pandemic period (*β* = −0.04; 95% CI: −0.24, 0.15; *p* = 0.654), and in the mid-pandemic (β = −0.12; 95% CI: −0.03, 0.07; *p* = 0.216), as compared to pre-pandemic time period (Table 3).

**Table 3:** Magnitude of change in unhealthy food consumption during the pandemic as compared to pre-pandemic period.

Time period	Unhealthy food consumption			
	Sugary food^a,b,c^		Convenience/fast food ^a,b,d^	
	Coefficient (*p*-value)	95%CI	Coefficient (*p*-value)	95%CI
Pre-pandemic		REFERENCE		
Early pandemic	**−0.29 (0.032)**	**−0.56, −0.02**	−0.04 (0.654)	−0.24, 0.15
Mid pandemic	**−0.51 (<0.001)**	**−0.76, −0.25**	−0.12 (0.216)	−0.3, 0.07

Bold indicates statistical significance; ^a^Interaction models adjusted for child race/ethnicity, beneficial food consumption; ^b^Mixed effects linear regression; ^c^Includes consumption of sweetened fruit drinks and teas, soda, sport drinks, sugary cereals, and frozen desserts in the previous week; ^d^Includes consumption of fried potatoes, potato chips, heat and serve food, and food from restaurant in the previous week.

### Association between unhealthy food consumption and household socioeconomic disadvantage

Table 4 examines differences in unhealthy food consumption across household socioeconomic disadvantage categories using data pooled across the pandemic, i.e., without reference to time period. Overall, we noticed that lower unhealthy food consumption among those who were more disadvantaged. Pooled across study periods, sugary food and convenience/fast food consumption appears to be highest in the low socioeconomic disadvantage group (reference group, Table 4). As compared to the low socioeconomic disadvantage group, sugary food consumption was significantly lower in the medium disadvantage group (*β* = −0.2; *p* = 0.036, 95% CI: −0.38, −0.013), and in the high socioeconomic disadvantage group (*β* = −0.16; *p* = 0.06, 95% CI: −0.33, −0.006), after adjusting for confounders. As compared to the low socioeconomic disadvantage group, convenience/fast food consumption was lower in the medium disadvantage group (*β* = −0.28; *p* = <0.001, 95% CI: −0.48, −0.20) and in the high disadvantage group (*β* = −0.34; *p* = <0.001, 95% CI: −0.41, −0.15), after adjusting for confounders.

**Table 4:** Cross-sectional associations of unhealthy food consumption with household socioeconomic disadvantage (pooled data from 2018 to 2022).

Household socioeconomic disadvantage	Unhealthy food consumption			
	Sugary food^a,b,c^		Convenience/fast food^a,b,d^	
	Coefficient (*p*-value)	95%CI	Coefficient (*p*-value)	95%CI
Low		REFERENCE		
Medium	**−0.2 (0.036)**	**−0.38, −0.013**	**−0.28 (<0.001)**	**−0.48, −0.20**
High	−0.16 (0.060)	−0.33, −0.006	**−0.34 (<0.001)**	**−0.41, −0.15**

Bold indicates statistical significance; ^a^Interaction models adjusted for child race/ethnicity, beneficial food consumption; ^b^Mixed effects linear regression; ^c^Includes consumption of sweetened fruit drinks and teas, soda, sport drinks, sugary cereals, and frozen desserts in the previous week; ^d^Includes consumption of fried potatoes, potato chips, heat and serve food, and food from restaurant in the previous week.

### Interaction effect of household socioeconomic disadvantage and time period on unhealthy food consumption

The post-regression estimates from the adjusted interaction model show that the socioeconomic differences in convenience/fast food consumption disappeared during the mid-pandemic phase (Table 5).The significant association between convenience/fast food consumption and household socioeconomic disadvantage was seen in pre-pandemic (pairwise estimate low vs. high socioeconomic disadvantage group: contrast = 0.33, *p* ≤ 0.001; low vs. medium socioeconomic disadvantage group: contrast = 0.31, *p* ≤ 0.001); and mid-pandemic period (pairwise estimate low vs. high socioeconomic disadvantage group: contrast = 0.27, *p* ≤ 0.001; low vs. medium socioeconomic disadvantage group: contrast = 0.22, *p* ≤ 0.001) time periods.

**Table 5:** Post regression estimates (pairwise comparison of effects) from the adjusted^a^ interaction model.

Time period	Household socioeconomic disadvantage groups	Unhealthy food consumption	
		Sugary food^b,c^	Convenience/fast food^b,d^
		Contrast (*p*-value)	
Pre-pandemic	Medium vs. High	0.01 (0.941)	0.02 (0.84)
	Low vs. High	0.2 (0.054)	**0.33 (<0.001)**
	Low vs. Medium	0.19 (0.09)	**0.31 (<0.001)**
Early pandemic	Medium vs. High	−0.36 (0.09)	−0.47 (0.782)
	Low vs. High	0.34 (0.139)	0.06 (0.713)
	Low vs. Medium	−0.4 (0.166)	0.53 (0.215)
Mid-pandemic	Medium vs. High	0.17 (0.438)	0.05 (0.09)
	Low vs. High	0.13 (0.55)	**0.27 (<0.001)**
	Low vs. Medium	−0.04 (0.867)	**0.22 (<0.001)**

Bold indicates statistical significance; ^a^Interaction models adjusted for child race/ethnicity, beneficial food consumption; ^b^Mixed effects linear regression; ^c^Includes consumption of sweetened fruit drinks and teas, soda, sport drinks, sugary cereals, and frozen desserts in the previous week; ^d^Includes consumption of fried potatoes, potato chips, heat and serve food, and food from restaurant in the previous week.

## Discussion

Periods of instability can cause significant changes in dietary behaviors. Although evidence of the short-term impacts of the COVID-19 pandemic has been explored by studies across the globe, there is little evidence of the long-term effects in the years following the pandemic. Our study investigated serial cross-sectional changes in unhealthy food consumption over the course of the pandemic. We observed a significant minor decrease in sugary food consumption during the early and mid-pandemic phases. In regard to convenience/fast food consumption, we observed a slight increase in consumption from the pre- to early pandemic, followed by a decrease to the mid-pandemic.

These results only partially align with the results from other national (33, 39–41) and international studies (9, 18, 42, 43); a majority of other studies that collected data in the first months of the pandemic (March–April 2020) report contradictory results (15–18). A cross-sectional study conducted by Adams et al. (20) reported a decrease in sweets and dessert consumption among approximately one-third of food-insecure families (20). A study conducted in Greece among secondary school students also reported a decrease in sweets and sugary drinks (42). We hypothesize that this may be partly attributed to the time of data collection. In a longitudinal study that examined changes in home food availability, it was noted that although about one-third of the sample reported an increase in desserts at home in May 2020, a decrease in the same was noticed in 30% of the families in September 2020 (33). As the data for our study was collected in the fall of each year, it is possible that “panic-buying” and “comfort food” purchases had subsided, and that the children had already adapted to their new lifestyle (33, 44). Another reason could also be the pandemic-related waivers in the implementation of government nutrition programs. For instance, state agencies reported that utilization of the COVID-19 Child Nutrition nationwide waivers improved the implementation of the National School Lunch Program (NSLP), Summer Food Service Program, and Child and Adult Care Food Program (45). Similarly, Adams et al. (46) noted that in 2021, parents who participated in child tax credit expansion reported a significant decrease in children’s consumption of added sugar and sugar-sweetened beverages (46). In addition to this, innovative implementation of health promotion programs such as non-profit–for-profit partnerships (47) and direct to consumer models (48), have been proven effective to improve access to fresh food in times of crises (49). It is important to evaluate the public health impact of pandemic-related policies and novel solutions, introduced during a disaster, on the behaviors of individuals in different economic strata.

We also observed heterogeneity in unhealthy food consumption among children, by socioeconomic disadvantage levels. There were significantly lower levels of convenience/fast food and sugary food consumption among those who were most disadvantaged. Of note, this sample was homogenously lower income given that one of the eligibility criteria for participating in Brighter Bites is that the school composition is >75% of the children receive free/reduced lunch program. However, even within our lower income sample, we saw differences by SES disadvantage. Previous studies have reported discrepancies in the association between socioeconomic status and eating behaviors. A study that examined family-affluence-related inequities in adolescent food consumption among 41 countries reported that in some countries, adolescents from the lowest family affluence consumed fewer fruits, vegetables, sweets, and chocolates (50). The low budget constraint and limited ability to buy food of any kind may explain this result (18, 50). Notably, the socioeconomic disadvantage measure in our study included multiple components. Previous studies report that children’s reduced consumption of healthy food was associated with mothers’ higher education and/or a full-time employment status (51). Parents who work full-time or multiple jobs may face time constraints in meal planning, preparation, and consequently in providing healthier food for their children (52, 53). However, we noticed a lack of socioeconomic heterogeneity in convenience/fast food consumption during early pandemic period. This may be attributed to the relatively uniform impact of the early pandemic period experiences across the disadvantaged groups of the low-income sample (54). Food supply chain disruptions and restaurant closures might have minimized differences in fast-food consumption since fewer families, regardless of their specific level of disadvantage, could easily obtain these foods. These findings underscore the critical need to develop sustainable and resilient food systems that ensure reliable access to healthy food options for all socioeconomic groups, particularly in times of crisis. It is imperative to examine multiple barriers to healthy food access that arise during and after a disaster, using comprehensive and nuanced measures (49). Moving beyond physical access to food, studies have also recorded the urgency of investigating other factors such as social accessibility, cultural acceptability, and food agency during a crisis and addressing these factors through community cross-sectoral collaboration (49, 55).

Our study was novel in examining the changes in unhealthy food consumption and its association with household socioeconomic disadvantage through the course of a public health crisis. Using multi-site data, the external validity of the study is strengthened. The serial cross-sectional nature of our data, along with access to pre-pandemic data helped us investigate changes over time, which is an added strength to this study. However, the validity of the study results may have been compromised due to multiple reasons. The results may have limited generalizability to other populations across the country, a. since those who participated in our study may have been in extreme need of help during the COVID-19 pandemic, b. a majority of our participants belonged to the Mexican/Hispanic/Latino race ethnicity. As this ethnic group exhibits a collectivist cultural lifestyle, the home environment findings reported may not be generalizable to individualistic group. The study may also be subjected to selection bias since study surveys were available only to those with digital access. The data may also be subjected to social desirability bias due to the self-reporting nature of the surveys. Finally, low response rate could have led to non-random loss of data.

## Conclusion

Our findings provide valuable insights into the varied impact of an unprecedented crisis on unhealthy food consumption of children from low-income families at different levels of socioeconomic disadvantage. The results can serve as a benchmark for community or government organizations to design tailored policies/intervention strategies that address the specific challenges faced by underserved communities. Careful investigation is required in qualifying a community as economically vulnerable to disasters or economic shutdowns. Further research needs to be conducted to understand the nuances of socioeconomic dynamics on the diet behaviors of families-in-need in the post-pandemic setting.

## Data Availability

The data analyzed in this study are not publicly available. Data may be made available upon reasonable request to the corresponding author. Requests to access these datasets should be directed to Nivedhitha.pp@gmail.com.
